# The comparative diagnostic and therapeutic application value of FAPI PET/CT and ^18^F-FDG PET/CT in oncology

**DOI:** 10.3389/fonc.2026.1751727

**Published:** 2026-01-28

**Authors:** Yongqi Yao, Jiawei Zhao, Jingjing Xiao, Yujing Chen, Xiaotong Guo, Jingyi Zhang, Peitao Wu, Lixuan Liu, Juzheng Zhang, Jinfeng Yang, Chunrong Chen, Jiamin Jin, Bo Ge

**Affiliations:** 1 Department of Urology, The Second Affiliated Hospital of Guilin Medical University, Guilin, Guangxi, China; 2 Key Laboratory of Tumor Immunology and Microenvironmental Regulation, Guilin Medical University, Guilin, Guangxi, China; 3 Guangxi Health Commission Key Laboratory of Tumor Immunology and Receptor-Targeted Drug Basic Research, Guilin Medical University, Guilin, Guangxi, China; 4 Department of Limb Trauma and Hand Surgery, Affiliated Hospital of Guilin Medical University, Guilin, Guangxi, China

**Keywords:** comparative study, FAPI, FDG, molecular imaging, PET/CT, precise diagnosis, tumor imaging

## Abstract

Molecular imaging has become central to oncologic diagnosis and therapy assessment. ^18^F-fluorodeoxyglucose positron emission tomography/computed tomography (^18^F-FDG PET/CT) is widely implemented, yet performance is attenuated in tumors with low glycolytic activity or in sites with high physiological uptake. Small-molecule fibroblast activation protein inhibitors (FAPI) enable high-contrast imaging of cancer-associated fibroblasts within the tumor stroma, offering rapid clearance and favorable biodistribution. This review synthesizes clinical and preclinical evidence comparing FAPI PET/CT with^18^F-FDG PET/CT across solid tumors. Head-to-head analyses indicate superior or complementary lesion conspicuity for FAPI in pancreatic ductal adenocarcinoma and colorectal cancer (CRC) —especially peritoneal and nodal disease—and context-dependent comparability in breast and head-and-neck cancers. Across studies, FAPI demonstrates higher tumor-to-background ratios and improved detection of small or low-FDG-avid lesions, with variable downstream effects on staging and management. We delineate disease-specific scenarios in which multi-tracer strategies may optimize diagnostic yield and propose a framework for integrating FAPI into precision imaging pathways. Priority areas include prospective, adequately powered trials; harmonized acquisition and quantification protocols; and evaluations of cost-effectiveness and theranostic implications.

## Introduction

**Figure 1:**
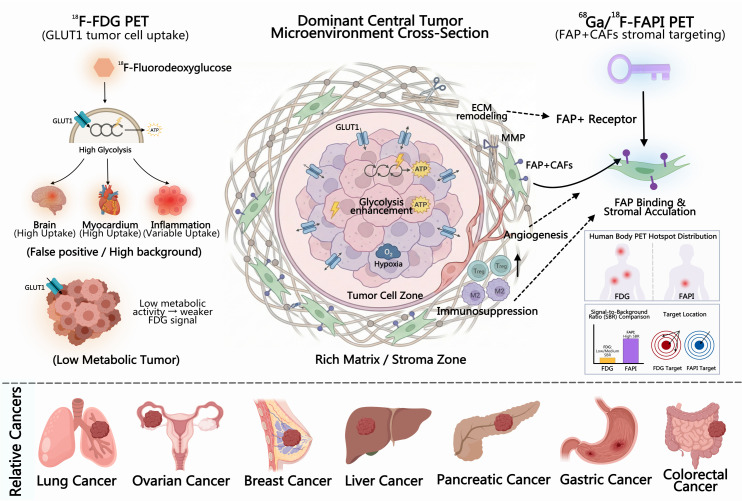
Schematic comparison of imaging mechanisms between^18^F-FDG and FAPI in tumor detection.This picture created by MedPeer (https://www.medpeer.cn/).

The multimodal integration of positron emission tomography (PET) and computed tomography (CT) allows for simultaneous functional and molecular as well as anatomical evaluation in a single examination, facilitating the transition from research to routine clinical oncology practice (1). By visualizing metabolic activity, receptor expression, and microenvironmental changes, PET/CT supports staging and restaging, assessment of treatment response, prognostication, and image-guided therapeutic planning (2). Traditional morphology-based imaging may fail to detect small lesions, overlook inter- and intra-tumor heterogeneity, and perform suboptimally in assessing early biological response, whereas PET/CT addresses these limitations through molecular readouts (3). ^18^F-fluorodeoxyglucose (^18^F-FDG) remains the cornerstone tracer, supported by globally standardized clinical frameworks (4). Its mechanism—glucose transporter (GLUT)-mediated uptake and hexokinase-mediated trapping—enables sensitive detection in glycolytically active tumors (5, 6). Nonetheless, non-specific uptake in inflammation or infection, high physiological background activity, and tumors with low-grade or mucinous histology can lead to false-positive and false-negative results, thereby reducing the accuracy of staging and treatment response assessment, particularly in head and neck, gastrointestinal, pancreaticobiliary, and gynecologic malignancies (7, 8). In recent years, molecular imaging strategies targeting the tumor microenvironment (TME) have advanced rapidly. The fibroblast activation protein (FAP), abundantly expressed on cancer-associated fibroblasts (CAFs) across numerous solid tumors, plays a pivotal role in key biological processes such as extracellular matrix remodeling, angiogenesis, epithelial–mesenchymal transition, and immune suppression. Through these functions, FAP has emerged as a compelling target for both diagnostic imaging and therapeutic intervention within the TME (9). Small-molecule fibroblast activation protein inhibitor (FAPI) probes demonstrate rapid accumulation within tumor tissues and efficient clearance from non-target organs, yielding high tumor-to-background ratios (TBR) alongside favorable *in vivo* pharmacokinetic and dosimetric characteristics. These attributes present a promising approach to overcoming the major limitations associated with ^18^F-FDG imaging (10, 11). The clinical translation of FAPI PET/CT has progressed rapidly, with a range of ^68^Ga-labeled and next-generation ^18^F-labeled FAPI derivatives (e.g., FAPI-04, FAPI-46, FAP-2286, FAPI-RGD) demonstrating superior lesion detection rates and improved tumor-to-background ratios (TBRs) compared to ^18^F-FDG across multiple cancer cohorts. These tracers also exhibit complementary diagnostic value in specific tumor types or clinical contexts (12). Compared with FDG, which depends on glucose metabolism and GLUT1-mediated transport, FAPI PET/CT provides superior specificity and enhanced contrast in detecting tumor microenvironment alterations, including extracellular matrix remodeling, angiogenesis, and immunosuppressive conditions (**Figure 1**).

Building on this background, this review aims to systematically compare FAPI PET/CT and ^18^F-FDG PET/CT in terms of biological targets, imaging kinetics and dosimetry, lesion detection rates, SUV/TBR metrics, staging and restaging accuracy, and their impact on clinical decision-making. Particular emphasis is placed on evaluating the evidence across distinct tumor types—including pancreatic cancer, gastric cancer, biliary tract tumors, mucinous and low-grade malignancies, head and neck squamous cell carcinoma, breast cancer, and CRC—as well as in specific clinical contexts such as postoperative or postradiotherapy settings, coexisting inflammation/fibrosis, and resectability assessment. This analysis outlines the strengths and potential limitations of each imaging modality. Through comprehensive evaluation, we seek to define the clinical scenarios in which FAPI or ^18^F-FDG should be preferentially or concurrently utilized to establish more personalized and cost-effective molecular imaging strategies.

## Understanding the science behind FAPI PET/CT and the evolution of its tracers

FAP, a tumor-microenvironment antigen, exhibits high enrichment on CAFs while maintaining minimal expression in normal tissues. The development of quinoline-based small-molecule FAP inhibitors (FAPIs), such as FAPI-02 and FAPI-04 in 2018, facilitated ^68^Ga/^18^F radiolabeling and enabled rapid, high-contrast PET/CT imaging with favorable pharmacokinetic and dosimetric profiles (13). Initial investigations demonstrated high uptake and superior lesion-to-background contrast across multiple solid tumors, with performance exceeding that of ^18^F-FDG in specific clinical scenarios—such as improved tumor-to-background ratio and TNM upstaging in CRC, alongside heightened sensitivity and TBR in ovarian peritoneal metastases (14). For clinical deployment ^68^Ga-FAPI has been rapidly adopted due to the availability of ^68^Ge/ ^68^Ga generators, straightforward radiolabeling synthesis, low physiological background activity, and robust tumor uptake across diverse malignancies (15). While ^18^F-labeled tracers, such as FAPI-74, broaden accessibility through centralized production, they have also demonstrated a significant impact on clinical management in specific patient cohorts (16). These findings support disease-specific head-to-head comparisons that emphasize detection performance, SUV/TBR, and the impact on clinical decisions.

## The application of FAPI-PET/CT in malignant tumors

### Respiratory system neoplasms

#### Lung cancer

Lung cancer remains the foremost cause of cancer-related mortality globally. Robust evidence indicates that low-dose computed tomography (LDCT) screening in high-risk populations significantly reduces lung cancer–specific mortality (17). FAPI PET/CT visualizes fibroblast activation protein (FAP)—expressed on CAFs—and augments conventional imaging by mapping stromal activation in the tumor microenvironment. Owing to its high image contrast and typically optimal lesion delineation at approximately one hour post-injection, FAPI PET/CT offers a clinically practical platform for precise tumor staging and definition of thoracic target volumes (18). At the lesion level, prospective studies indicate that ^18^F-FAPI exhibits superior sensitivity (99% vs. 87%), specificity (93% vs. 79%), accuracy (97% vs. 85%), and negative predictive value (97% vs. 70%) compared to ^18^F-FDG across primary tumors, nodal involvement, and distant metastases, while maintaining a comparable positive predictive value—underscoring its enhanced utility for ruling out disease during whole-body staging (19). Meta-analyses similarly demonstrate superior aggregated sensitivity for metastatic lesions (0.99 vs. 0.77), whereas sensitivity for primary tumors is comparable between FAPI and FDG (0.98 vs. 0.99). Semi-quantitatively, the target-to-background ratio (TBR) is lower for FAPI than FDG in primary lung tumors (25.3 ± 14.0 vs. 32.1 ± 21.1) but higher in metastatic lymph nodes and bone metastases (7.5 ± 6.6 vs. 5.9 ± 8.6; 8.6 ± 5.4 vs. 4.3 ± 2.3), suggesting enhanced contrast advantages particularly in osseous disease (20). Pathologic correlation analyses further confirmed significantly higher ^18^F-FAPI uptake in mediastinal and hilar lymph nodes (SUV~max~ 10.87 ± 7.29 vs. 6.08 ± 5.37), yielding superior diagnostic performance compared to FDG, with respective sensitivity, specificity, accuracy, PPV, and NPV values of 84%, 92%, 90%, 84%, and 92% versus 71%, 67%, 69%, 52%, and 83%. Importantly, for subcentimeter nodes (<10 mm), ^18^F-FAPI demonstrated enhanced specificity, accuracy, and PPV (96%, 93%, and 77% vs. 72%, 73%, and 33%), underscoring its enhanced reliability in identifying small nodal metastases (20). In a prospective perioperative cohort, an ^18^F-FAPI SUV_max threshold of 6.2 effectively discriminated benign from metastatic lymph nodes (NPV 93.8%; PPV 87.5% in the absence of calcification or high-density features); integration of dual-tracer imaging improved nodal staging accuracy to 83% and led to changes in clinical management for 29 patients (21). Analysis of real-world NSCLC cohorts reveals comparable primary tumor SUV_max/TBR, yet demonstrates enhanced detection of nodal and osseous metastases with FAPI, leading to cancer stage reclassification in approximately 11% of patients (22). A subsequent single-center prospective study similarly identified a higher prevalence of suspicious nodal, osseous, and pleural lesions using FAPI compared to FDG (23). To systematically assess the performance differences between FAPI and ^18^F-FDG in oncological imaging, we have synthesized key parameters from current representative studies, enabling a comprehensive comparison of their diagnostic accuracy, tumor targeting efficiency, and clinical applicability (**Table 1**). Compared with ^18^F-FDG, FAPI-PET/CT demonstrates superior sensitivity, specificity, and tumor-to-background ratio (TBR) in N/M staging of lung cancer, particularly for mediastinal and hilar lymph nodes as well as bone, brain, and pleural metastases, while maintaining a comparable overall detection rate for primary lesions. Emerging evidence also suggests that FAPI-PET/CT may reflect the immune microenvironment, such as PD-L1 expression, thereby offering the potential to improve the accuracy of staging and inform more precise treatment decision-making.

**Table 1:** Summary of key parameter comparisons (FAPI vs FDG, Lung Cancer).

Indicators/Scenarios	FAPI	FDG	References
The sensitivity of all lesion levels	99%	87%	(19)
The specificity of all lesion levels	93%	79%	(19)
The accuracy of all lesion levels	97%	85%	(19)
Mean TBR of primary lung tumor	25.3	32.1	(19)
Mean TBR of metastatic lymph nodes	7.5	5.9	(19)
Mean TBR of bone metastasis	8.6	4.3	(19)
Mean TBR of brain metastases	314.4	1	(23)
The comprehensive sensitivity of mediastinal/hilar lymph nodes	84%	71%	(20)
The comprehensive specificity of mediastinal/hilar lymph nodes	92%	67%	(20)
Specificity of small lymph nodes (< 10 mm)	96%	72%	(20)
Accuracy of small lymph nodes (< 10 mm)	93%	73%	(20)
Accuracy rate of N stage integration	83%(FAPI+FDG)	Non-direct comparison items	(21)
The difference in SUV_max/TBR of the primary lesion	No significant differences were observed	No significant differences were observed	(24)
Systemic metastasis detected (LN/bone/pleura)	More	less	(24)
Combined sensitivity of metastatic foci	0.99	0.77	(25)
PD-L1 correlation (trend)	Positive correlation	Benign inflammatory interference	(22)
Imaging Timing and Dose	1 hour comparison is the best; low effective dose	Benign inflammatory interference	(18)
Benign inflammatory interference	Possibly positive	Possibly positive	(26)

### Tumors of the female reproductive system

#### Ovarian cancer

Globally, ovarian cancer (OC) is the seventh most commonly diagnosed malignancy in women and ranks as the eighth primary cause of female cancer-related deaths (27). In epithelial ovarian cancer (EOC), conventional imaging and ^18^F-FDG PET/CT often fail to detect small metastatic lymph nodes and peritoneal deposits, highlighting the necessity for tracers with a high tumor-to-background ratio (TBR) to improve staging accuracy and surgical strategy. In a prospective comparative trial, [68Ga]Ga-FAPI-04 and ^18^F-FDG demonstrated similar diagnostic performance at the patient level for identifying primary tumors (sensitivity: 93.9% vs 90.9%; specificity: 80.0% vs 80.0%; accuracy: 92.1% vs 89.5%); however, FAPI imaging achieved a markedly higher tumor-to-liver ratio (TLR; median: 15.95 vs 4.94; P < 0.001), indicative of superior lesion contrast due to reduced background uptake (28). Notably, physiological ovarian FDG uptake fluctuates according to the menstrual cycle, which can compromise diagnostic specificity; in contrast, FAPI uptake exhibits no such cyclical variation (29). In pathology-correlated studies of peritoneal seeding, FAPI demonstrates consistently superior patient-level sensitivity compared to FDG (97.5% vs. 75.9% and 96.8% vs. 83.0%; both P < 0.001), in addition to significantly improved semi-quantitative parameters (SUV_max: 17.31 vs. 13.68, P = 0.026; TLR: 23.81 vs. 5.39, P < 0.001; PCI: 15 vs. 11, P < 0.001) (28). In patients with platinum-sensitive recurrent disease, FAPI imaging demonstrates a substantial improvement in both sensitivity and accuracy (96.30% vs 49.07% and 97.40% vs 63.87%, respectively), an effect primarily driven by enhanced tumor-to-background ratio rather than by differences in absolute SUV_max (30). Lesion-distribution analyses further indicate enhanced detection of peritoneal and pleural metastases (e.g., 9/9 vs. 5/9) and occasional upstaging, underscoring the additional value of this approach for systemic staging (31). For the assessment of diaphragmatic and upper abdominal dissemination, FAPI-PET/MR imaging benefits from a low hepatic background, yields peritoneal carcinomatosis index estimates that align more closely with surgical observations, and enhances the prediction of incomplete cytoreduction (sensitivity: 91.67%; specificity: 64.29%) (32). Consistent systematic evidence across gynecologic malignancies reveals a superior pooled sensitivity for detecting peritoneal cancer with FAPI (approximately 0.98) compared to FDG (0.71), accompanied by reduced background signal and an increased tumor-to-background ratio (33). Collectively ^68^Ga-FAPI PET/CT provides enhanced contrast and superior sensitivity for detecting peritoneal implants and specific nodal metastases in ovarian cancer (OC), whereas FDG retains utility for metabolic characterization; a dual-tracer approach may thus more comprehensively capture complementary molecular–mesenchymal phenotypes and refine clinical decision-making (28, 32). Clinically, FAPI-guided staging resulted in upstaging of disease in a substantial proportion of patients (14.3% in newly diagnosed and 33.3% in recurrent EOC), which consequently altered clinical management in 10.7% and 19.0% of cases, respectively. These findings support the preferential application of FAPI imaging for evaluating suspected peritoneal seeding, assessing resectability, and monitoring recurrence, although definitive recommendations await larger prospective trials investigating long-term patient outcomes (28). To systematically present the comparative performance of FAPI and ^18^F-FDG across key parameters in ovarian cancer, this article summarizes and synthesizes the available research data (**Table 2**). Current head-to-head and meta-analytic evidence indicates that ^68^Ga-FAPI PET/CT demonstrates a higher detection rate and significantly improved tumor-to-background ratio (TBR) compared to ^18^F-FDG PET/CT in the evaluation of ovarian cancer, particularly for peritoneal implants and selected lymph node metastases. In various clinical scenarios, the SUV_max values obtained with FAPI are at least comparable to, and in some cases higher than, those of FDG. This contributes to more accurate staging and enhanced clinical decision-making potential.

**Table 2:** Summary of key parameter comparisons (FAPI vs FDG, Ovarian Cancer).

Indicators/Scenarios	FAPI	FDG	References
Primary lesion sensitivity (patient level)	100%(11/11)	100%(11/11)	(29)
Primary lesion sensitivity (lesion/preoperative)	100%(14/14)	90%(9/10)	(29)
Sensitivity of recurrent lesions (lesion level)	96.30%	49.07%	(30)
Accuracy of recurrent lesions (lesion level)	97.40%	63.87%	(30)
Peritoneal metastasis sensitivity (patient level)	95.00%	83.33%	(29)
Peritoneal metastasis sensitivity (region/focus)	90.16%	60.66%	(29)
Detection rate of peritoneal metastasis (comparison within the study)	96.80%	83.00%	(28)
Peritoneal/pleural metastasis sensitivity (lesions, small sample)	100%(9/9)	56%(5/9)	(31)
Peritoneal metastasis SUVmax (mean/median)	17.31	13.68	(28)
Peritoneal metastasis TBR (median)	7.55	2.7	(29)
Lymph node metastasis sensitivity (lesion/pathological basis)	80.60%	61.30%	(28)
Lymph node-specific (lesion/pathological benchmark)	95.10%	96.10%	(28)
The maximum SUV of lymph nodes (posterior abdominal/above the diaphragm)	8.72/6.39	6.56/4.20	(28)
Mean ± SD of TBR in lymph nodes	11.13 ± 9.59	3.24 ± 3.12	(30)
The overall TBR (mean ± SD) of recurrent lesions	7.50 ± 6.42	3.19 ± 2.96	(30)
Tumor burden (Eisenkop score)	27	16	(30)
Change in treatment strategy (recurrence cohort)	17.24% of the patients had their treatment adjusted based on FAPI information.	–	(30)

Figure caption example:-:No data.

### Tumors of the mammary system

#### Breast cancer

Despite notable advances in cancer research, breast cancer remains a significant global health challenge and continues to be a central focus of biomedical investigation (34). Fibroblast activation protein (FAP), which is highly expressed in CAFs of the breast tumor stroma, permits FAPI-based tracers to achieve elevated tumor uptake alongside a low physiological background. This results in an enhanced tumor-to-background ratio (TBR) and improved lesion conspicuity, especially for sub-centimeter lesions and areas with minimal inflammatory activity (35). In a prospective head-to-head comparison involving 34 newly diagnosed patients, FAPI exhibited significantly higher SUV_max and tumor-to-background ratio (TBR) in primary lesions compared to FDG (SUV_max: 11.06 ± 5.48 vs. 8.33 ± 6.07; TBR: 15.32 ± 10.33 vs. 8.25 ± 5.51; both statistically significant). Moreover, FAPI SUV_max showed a positive correlation with pathological grade and clinical stage, while lesion-level detection rates were 100% (50/50) for FAPI versus 96% (48/50) for FDG (36). In a separate comparative analysis employing direct TBR quantification, primary lesions exhibited a significantly higher median TBR with FAPI than with FDG (14.8 versus 6.3, p = 0.002) (35). For the assessment of lymph node metastasis, FAPI demonstrated superior specificity and diagnostic accuracy compared to FDG, achieving a higher overall N-staging accuracy (91.2% vs 73.5%) and enhanced detection of axillary N0 status (85.7% vs 42.9%) (36). In low-FDG-avid disease subgroups, FAPI imaging can detect additional nodal involvement in the axillary, supraclavicular, and mediastinal regions, including subcentimeter nodes that are solely FAPI-positive, with potential implications for disease upstaging (37). The comparatively low physiological uptake of FAPI in normal bone and brain tissue results in higher target-to-background ratios and superior visualization of osseous and cerebral metastases compared to FDG (35). In cases of liver metastases, while SUV_max values may be comparable, FAPI significantly enhances the tumor-to-background ratio and enables the detection of lesions not visible on FDG-PET, owing to its minimal physiological uptake in normal liver tissue (35, 38). Paired multi-cohort analyses indicate that FAPI and FDG exhibit comparable patient-level diagnostic accuracy in breast cancer, albeit with limitations due to small sample sizes, while regional sensitivity and accuracy are largely similar. Nevertheless, a modestly lower negative predictive value (NPV) observed with FAPI may indicate underlying heterogeneity among biological subtypes and lesion stages (39). Notably, FAPI imaging can yield false-positive results in the context of fibrotic or granulomatous lesions, highlighting the necessity for comprehensive clinical correlation and multimodal imaging integration (40). Collectively, FAPI imaging represents a valuable adjunct to FDG for initial breast cancer staging, particularly in confirming axillary N0 status, refining evaluation in cases with low FDG avidity, and improving the detection of brain, bone, and hepatic metastases (35–37). In summary, FAPI PET/CT demonstrates higher SUV_max and tumor-to-background ratio (TBR) compared to ^18^F-FDG in both primary breast cancer lesions and multi-organ metastases, particularly in axillary N0 staging, low FDG-avid subtypes, and metastases involving the liver, bone, and brain. Furthermore, FAPI PET/CT delivers at least non-inferior and potentially superior diagnostic performance.

### Solid tumors of the digestive system

#### Liver cancer

Primary liver cancer is the fourth leading cause of cancer-related deaths worldwide. Histologically, liver cancer is primarily categorized into two major subtypes: hepatocellular carcinoma, which accounts for approximately 75% of all cases, and ICC, representing about 15% of all liver cancers (41). While ^18^F-FDG PET, which reflects glucose metabolism, typically demonstrates low or heterogeneous uptake in well-to-moderately differentiated hepatocellular carcinoma (HCC) and certain ICC subtypes, its diagnostic utility is constrained by high physiological liver activity, resulting in a suboptimal tumor-to-background ratio (TBR). Conversely, FAPI imaging targets CAFs in the tumor microenvironment, leading to low background activity in normal liver parenchyma and elevated uptake in fibrosis-rich or stroma-dense tumors, thereby enhancing lesion detectability. However, the potential for false-positive findings due to benign inflammatory processes necessitates correlative interpretation with anatomical imaging and clinical context (42). In a prospective observational cohort (n=44), ^18^F-FAPI-04 exhibited superior sensitivity relative to ^18^F-FDG in detecting malignant liver lesions (84.6% [33/39] vs 76.9% [30/39]), albeit with reduced specificity (60% [3/5] vs 100% [5/5]), accompanied by significantly elevated SUV_max and tumor-to-background ratio (TBR) values (43). A head-to-head comparative study, stratified by histology, revealed a superior overall sensitivity for FAPI (96% [22/23]) compared with FDG (65% [15/23]), with specific improvements observed in both hepatocellular carcinoma (94% vs 69%) and ICC (100% vs 57%) (42). At the lesion level, FAPI demonstrated superior performance compared to FDG (85.7% vs. 57.1%), especially in detecting small lesions ≤2 cm (68.8% vs. 18.8%) and moderately to well-differentiated hepatocellular carcinoma (83.3% vs. 33.3%) (44). ^18^F-FAPI demonstrates a greater influence on the initial staging and therapeutic decision-making for hepatocellular carcinoma (HCC), as it enables the detection of more primary lesions and lymph node or peritoneal metastases, leading to T-stage upstaging. However, ^18^F-FDG retains certain advantages in assessing large vessel invasion and selected bone metastases (45). FAPI demonstrates significantly higher sensitivity and detection rates compared to FDG, particularly in early-stage or small-volume lesions and high-to-moderately differentiated hepatocellular carcinoma (HCC). However, its specificity is moderately lower than that of FDG due to inflammatory false-positive findings, necessitating the incorporation of multi-modal imaging and clinical evidence for accurate interpretation (43). The median SUV_max/TBR was also significantly higher with FAPI (13.61/5.55) than with FDG (4.24/1.17), a finding that extended to intrahepatic cholangiocarcinoma (TBR 6.95 vs. 1.49). Notably, FAPI-derived metrics increased progressively with worsening hepatocellular carcinoma differentiation, whereas FDG uptake failed to show clear stratification, underscoring the biological complementarity of the two imaging approaches (42). In mixed hepatocellular–cholangiocarcinoma, Ki-67 expression exhibited a positive correlation with FAPI-SUV_max (R = 0.603), and specific immunophenotypes were linked to elevated FAPI uptake, whereas no significant associations were observed for FDG-derived metrics. Mild hepatic fibrosis or steatosis may lead to a modest increase in FAPI background activity, thereby attenuating improvements in tumor-to-background ratio (TBR). In addition, the presence of inflammatory pseudotumors or dysplastic nodules can compromise diagnostic specificity, as illustrated by a reduction from 100% to 60% in certain cases (43). For whole-body staging, FAPI imaging identified a greater number of peritoneal and osseous lesions (e.g., peritoneum: 12 vs 4; bone: 43 vs 33), yet demonstrated no distinct superiority in the detection of pulmonary metastases, indicating complementary patterns of metastatic involvement (42). Collectively, FAPI demonstrates superior sensitivity, SUV_max, and tumor-to-background ratio in liver tumors, aligning with existing comparative data (46). Prospective data substantiate the utility of this approach for small-volume and well-to-moderately differentiated HCC, while FDG-PET may more effectively delineate specific invasive and metastatic characteristics; the integration of dual-tracer imaging strategies could enhance initial staging accuracy and refine management planning (44, 45). Therefore, FAPI-PET/CT should be prioritized in the initial staging, recurrence evaluation, and systemic metastasis characterization of HCC and ICCC, particularly in scenarios where MRI/CT findings are suspicious but FDG shows negative or low uptake, or when a high tumor-to-background ratio (TBR) is required for the assessment of small lesions or those dominated by interstitial reactions. For suspected inflammatory lesions or cases with significant liver fibrosis, complementary use of MRI, multiparametric CT, and clinical-pathological parameters is recommended to enhance diagnostic specificity.

#### Pancreatic cancer

Pancreatic cancer remains among the most lethal malignancies worldwide. According to the GLOBOCAN 2012 estimates, it accounts for more than 331,000 annual deaths, ranking as the seventh leading cause of cancer-related mortality in both men and women (47). In pancreatic cancer, predominantly pancreatic ductal adenocarcinoma (PDAC), FAPI-PET/CT, which targets the CAF-mediated stromal response, demonstrates consistently superior overall diagnostic performance compared to ^18^F-FDG PET/CT, which relies on glucose metabolism. Accumulating evidence from previous systematic reviews indicates that FAPI offers improved detection rates and enhanced quantitative contrast,as reflected by higher SUV and TBR values, across primary tumors, lymph node metastases, distant metastatic lesions, and peritoneal implants (48, 49). A meta-analysis focusing specifically on pancreatic ductal adenocarcinoma (PDAC), which included 7 studies and 322 patients, demonstrated a significantly higher pooled sensitivity for FAPI compared to FDG (0.99 [0.97–1.00] versus 0.84 [0.70–0.92]), while specificity remained comparable (0.84 [0.63–0.94] versus 0.85 [0.75–0.91]). The analysis also revealed a larger area under the curve (AUC) for FAPI (0.99 [0.98–1.00] versus 0.91 [0.88–0.93]). This superiority was consistent at both the patient and lesion levels, especially for nodal and distant metastases, and FAPI exhibited a higher SUV_max in primary lesions, with a mean difference of +6.47 (49). Consistent findings across multiple cohorts further validate the superior tumor-to-background ratio (TBR) achieved with FAPI, exemplified by enhanced contrast in liver metastases (e.g., TBR 5.7 ± 3.2 versus 3.2 ± 1.3), underscoring its utility in improving lesion detectability in low-contrast imaging scenarios (49, 50). Prospective head-to-head comparative studies (including those utilizing ^18^F-AIF-FAPI-74) reveal significantly lower background uptake (median SUVmean 0.8 versus 2.6) and superior tumor-to-background ratios, thereby enhancing the detection of small-volume nodal metastases and peritoneal implants and improving the certainty of staging evaluations (51). The superior imaging capabilities yield clinically significant impacts on staging and management: single-institution studies report nodal upstaging rates as high as 51.6% and alterations in treatment plans in up to 38.7% of cases, whereas pooled analyses reveal overall staging reclassification and management modification rates of 25% and 11.7%, respectively (49). In a prospective comparative study of ^18^F-FAPI-04 and FDG, TNM staging was upstaged in 14 out of 62 patients, highlighting the enhanced utility of FAPI for perioperative staging and the evaluation of resectability (51). Beyond staging, baseline imaging phenotypes, including the “distal pancreatitis” sign, can signal ductal obstruction, greater metastatic burden, and poorer prognosis; quantitatively, in a cohort of 51 patients, a FAPI-SUV_max threshold of 14.9 independently stratified overall survival (hazard ratio 8.877), suggesting that stroma-targeted tracer uptake intensity may reflect aggressive tumor biology and facilitate risk stratification (50). To systematically present the comparative performance of FAPI and ^18^F-FDG across key parameters in pancreatic cancer, this article summarizes and synthesizes the available research data (**Table 3**). Overall, FAPI-PET/CT demonstrates consistent advantages over FDG in sensitivity and image contrast for both primary and metastatic lesions in pancreatic cancer, while exhibiting comparable specificity. These characteristics suggest its potential to transform perioperative staging and recurrence monitoring workflows in pancreatic cancer management. Further validation in multicenter prospective trials is warranted to explore complementary strategies with FDG, such as delayed imaging and multi-tracer combinations, to optimize real-world clinical decision-making. Specifically, in pancreatic cancer, FAPI-PET/CT outperforms ^18^F-FDG in terms of sensitivity and tumor-to-background ratio (TBR) for detecting primary tumors, lymph node involvement, and distant metastases, without compromising specificity, thereby improving the accuracy of TNM staging and the formulation of treatment strategies.

**Table 3:** Summary of key parameter comparisons (FAPI vs FDG, Pancreatic Cancer).

Indicators/Scenarios	FAPI	FDG	References
Merged sensitivity	0.99(0.97–1.00)	0.84(0.70–0.92)	(49)
Specificity of combination	0.84(0.63–0.94)	0.85(0.75–0.91)	(49)
Area under the ROC curve	0.99(0.98–1.00)	0.91(0.88–0.93)	(49)
SUV_max of primary lesion (mean difference/weighted mean difference)	6.47	–	(49)
Primary lesion sensitivity (patient level)	1	0.94	(49)
Lymph node sensitivity (patient level)	0.96	0.66	(49)
Distant liver metastasis sensitivity (patient level)	1.00/1.00	0.75/0.80	(49)
Background SUV median	0.8(median)	2.6(median)	(51)
Primary lesion TBR (blood pool reference)	6.9 ± 2.5;4.4 ± 3.8	2.6 ± 1.3;4.0 ± 2.6	(49)
TBR of liver metastasis	5.7 ± 3.2	3.2 ± 1.3	(50)
Change in TNM staging (combined/single center upper limit)	25%; up to 51.6%	–	(49)
Clinical management changes (combined/single center upper limit)	11.7%; up to 38.7%	–	(49)
Individual studies on TNM upregulation	14/62(23%)	–	(49)
Prognostic threshold (SUV_max)	14.9(HR 8.877)	–	(50)

Figure caption example:-:No data.

#### Gastric cancer

Less than a century ago, gastric cancer was the most common malignancy in the United States and globally. Despite a significant decline in its global incidence over the past century, it remains a leading cause of cancer-related mortality worldwide (52). In contrast to ^18^F-FDG, FAPI enables superior tumor-to-background contrast in gastric cancer by specifically targeting CAFs, while exhibiting minimal physiologic uptake in the gastrointestinal tract, thereby allowing more precise disease staging and therapy-response evaluation (53–55). In prospective, head-to-head analyses of newly diagnosed cohorts, FAPI demonstrated a primary-tumor detection rate of 100%, compared with 50.0% for FDG; a separate prospective PET/MR study similarly revealed superior performance for FAPI (86.7% vs 60.0%) (56, 57). Quantitatively, FAPI demonstrates significantly higher radiotracer uptake and superior contrast, as evidenced by elevated median SUVmax (10.3 vs. 8.1) and tumor-to-background ratio (TBR; 11.6 vs. 5.8). These findings are corroborated by a separate prospective cohort, which reported a further increased TBR (11.9 vs. 3.2). Additionally, FAPI exhibits consistently higher tumor-to-liver ratios (TLR) across various anatomical regions (53, 56, 58). In the evaluation of lymph node metastasis, FAPI demonstrates enhanced sensitivity, specificity, and overall diagnostic performance. Nodule-based analysis revealed that the sensitivity of FDG is 54%, whereas FAPI achieves a significantly higher sensitivity of 79%. A retrospective cohort analysis further showed that FAPI exhibits an accuracy of 92.2%, sensitivity of 78.6%, and specificity of 96.0% for lymph node staging, all of which are either superior to or non-inferior to FDG. Notably, when validated against pathological/surgical findings, FAPI shows significantly higher specificity and positive predictive value compared to FDG (100.0% vs. 97.7%; 100.0% vs. 57.1%, respectively) (53, 58, 59). For peritoneal dissemination, intra-patient comparative analyses reveal a marked superiority of FAPI (detection rate 100% vs. 0%; mean SUVmax 10.0 vs. 2.4). Furthermore, a study published in EJNMMI documented a greater number of detected lesions and a higher tumor-to-background ratio (TBR) with FAPI (159 vs. 47 lesions; TBR 8.1 vs. 3.2). These findings are corroborated by systematic reviews and meta-analyses, which consistently report enhanced sensitivity and improved SUVmax and TBR values for the detection of peritoneal disease (55, 56, 58). FAPI demonstrates particular utility in mucinous adenocarcinoma and signet ring cell carcinoma (SRCC), tumor types that are frequently “metabolically cold” on FDG–PET imaging; comparative studies and case evidence in SRCC indicate that FAPI can detect lesions, including peritoneal deposits, that are not visualized by FDG (56, 60, 61). Nevertheless, FDG avidity may be diminished in early-stage malignancies and micro-foci characterized by limited volume or shallow infiltration, scenarios in which FDG-PET may retain diagnostic value and dual-tracer approaches could serve a complementary role (57, 60). For the early assessment of treatment response, a reduction of ≥52% in both %SUVmax and %TBR on FAPI imaging after a single cycle of neoadjuvant chemotherapy serves as a predictor of major pathological response (AUC 0.856–0.864; accuracy ~89.3%), with performance superior to that of FDG-derived parameters (54). Overall, FAPI-PET/CT demonstrates superior performance to FDG in detecting primary tumors, assessing peritoneal dissemination, and staging lymph node involvement, positioning it for broader application in radiotherapy target delineation and individualized therapeutic monitoring (53, 56, 58). In summary, FAPI-PET/CT demonstrates consistently superior performance compared to ^18^F-FDG-PET/CT in the detection of primary gastric cancer lesions, particularly in subtypes with low glucose metabolism and peritoneal metastases, as evidenced by improved imaging contrast—most notably tumor-to-background ratio (TBR). FAPI is capable of clearly visualizing signet ring cell carcinomas that exhibit negative or low FDG uptake, thereby influencing disease staging. The implementation of high-quality, standardized prospective studies, along with broader clinical adoption of FAPI, is anticipated to further improve its clinical accessibility and elevate its level of evidence.

#### Colorectal cancer

CRC now ranks as the fourth most common cause of cancer-related deaths globally, accounting for approximately 900,000 fatalities each year, the expansion of screening programs is intended to enhance early detection and lower both disease incidence and mortality (62, 63). By specifically targeting fibroblast activation protein (FAP) within the tumor stroma, FAPI demonstrates pronounced tumor uptake while exhibiting minimal physiological background signal in the intestinal lumen and peritoneal cavity, thereby offering a high-contrast platform for CRC staging and metastasis assessment (64). For the detection of primary lesions, head-to-head comparisons and meta-analyses reveal that FAPI and FDG exhibit comparable diagnostic performance (relative risk ≈ 0.99) (64, 65). Notwithstanding comparable SUVmax values (14.3 ± 8.6 vs. 15.4 ± 9.8, P = 0.604), FAPI demonstrates superior visualization of mucinous adenocarcinoma and signet ring cell carcinoma, achieving a significantly higher tumor-to-background ratio (64). Nodal evaluation demonstrates superior performance for FAPI, with surgically validated studies reporting sensitivities and specificities for lymph node detection as high as 90% and 100%, respectively, compared to 80% and 81.8% for FDG (66). The conclusion was further substantiated by lesion-level analyses, which indicated a significant advantage for FAPI (relative risk = 0.63, 95% confidence interval: 0.47–0.84) (65). Analysis of patient-level gastrointestinal cohorts further revealed that FAPI imaging achieved superior accuracy and sensitivity in determining nodal status compared to conventional methods (92.2% vs. 70.3% and 78.6% vs. 71.4%, respectively) (53). This finding is consistent with the heightened sensitivity observed in the digestive-tract subgroup (79% vs. 54%) (61). For distant metastases, the low hepatic background activity of FAPI may enhance the detection of small liver metastases; paired data in CRC revealed a greater number of liver lesions with FAPI compared to FDG (13 versus 7) (65). The paired CRC cohort demonstrated that FAPI identified more liver metastases (13 vs. 7) compared to FDG, indicating a potential clinical advantage in lesion detection. FAPI exhibited its most pronounced superiority in the detection of peritoneal metastases. The CRC study showed that FAPI not only detected a greater number of peritoneal lesions (107 vs. 45), but also yielded higher peritoneal cancer index (PCI) scores (median: 11 vs. 4). Findings from the recurrent CRC study further confirmed that FAPI outperformed FDG in both the detection of peritoneal metastases and PCI assessment, with a higher area under the ROC curve observed in the same patient cohort (67). Quantitative evaluation of peritoneal tumor burden using FDG-PET/CT is not recommended (68). Overall, FAPI enhances the assessment of metastatic burden and staging precision, can lead to TNM upstaging, and may influence therapeutic decision-making (61, 69). Using a mixed cohort of gastric and CRC as an example, FAPI demonstrates superior performance in detecting distant metastases, which enhances the accuracy of overall staging determination and consequently improves staging precision and clinical stratification (53). Quantitatively, FAPI generally yields superior tumor-to-background and tumor-to-liver ratios, whereas the maximum standardized uptake value (SUVmax) is frequently similar across different tracers (53, 64, 66) Registry data across various tumor types further indicate a superior overall diagnostic accuracy for CRC (39). From a biological perspective, these benefits are likely attributable to a high affinity for the CAF-rich tumor stroma, supporting the future incorporation of theranostic approaches in CRC (70). To systematically present the comparative performance of FAPI and ^18^F-FDG across key parameters in CRC, this article summarizes and synthesizes the available research data (**Table 4**). In conclusion, in CRC, FAPI-PET/CT demonstrates superior tumor-to-background ratios—particularly in the peritoneum and liver parenchyma with low background activity—and exhibits enhanced sensitivity for detecting peritoneal dissemination and lymph node metastases. It also shows greater diagnostic performance in mucinous and signet ring cell subtypes and is capable of identifying small-volume liver metastases. These advantages increase the likelihood of TNM stage upstaging and facilitate treatment strategy modifications. FAPI-PET/CT should be considered as a preferred imaging modality in clinical scenarios involving suspected peritoneal metastasis, ^18^F-FDG-negative or equivocal findings, mucinous histology, or recurrence restaging. FAPI-PET/CT offers the potential for more stable contrast enhancement and reproducible quantitative assessment.

**Table 4:** Summary of key parameter comparisons (FAPI vs FDG, Colorectal Cancer).

Detection rate of primary lesion (FAPI vs FDG)	Primary SUVmax (FAPI vs FDG)	Primary contrast (TBR/TLR, FAPI vs FDG)	Lymph node assessment (sensitivity/specificity/accuracy)	Peritoneal metastasis (detection/sensitivity)	Peritoneal contrast (SUVmax or TBR/TLR, FAPI vs FDG)	References
100% vs 100%	Lower vs. higher (P < 0.001)	higher(P=0.008)	90%/100%/95% vs 80%/81.8%/80.5%	FAPI was significantly superior, with a sensitivity of approximately 100% versus 55%.	TBR was significantly higher	(66)
100% vs 93.8%	14.3 ± 8.6 vs 15.4 ± 9.8(P=0.604)	19.0 ± 10.4 vs 11.8 ± 7.8(P=0.001)	87.5%/87.5%/87.5% vs 87.5%/75.0%/81.3%	Number of lesions 107 vs 45;PCI 11 vs 4(All P < 0.001)	SUVmax 9.0 ± 7.9 vs 2.6 ± 1.7;TBR 9.5 ± 4.4 vs 4.3 ± 2.5(All P<0.001)	(64)
100% vs 100%	10.3 vs 8.1(Median, P = 0.09)	13.3 ± 8.9 vs 8.2 ± 6.5(P<0.001)	-(CRC’s dedicated LN stratification is insufficient)	Lesion count: 159 vs 47 (more with FAPI)	TBR 8.1 vs 3.2(Median, P<0.001)	(71)
-(Exclusive to CRC)	-(Exclusive to CRC)	The TLR of all lesions was significantly higher.	Lower gastrointestinal LN, 79% vs 54% (sensitivity)	Higher (patient level)	Peritoneal TLR 7.8 vs 1.9;TBR 4.9 vs 3.0	(61)
-(Merged analysis)	-(Combined Analysis)	-(Combined Analysis)	78.6%/96.0%/92.2% vs 71.4%/70.0%/70.3%(Acc P = 0.002)	-(Individual CRC)	–	(53)
In the recurrence scenario, FAPI is higher	-(Original SUV)	-(Primary TBR)	-(quantitative)	FAPI is more sensitive, PCI is higher, and AUC is higher.	The peritoneum has higher contrast (qualitative)	(67)
-(Single column)	-(Single column)	The overall FAPI is higher	Overall sensitivity/accuracy, 98%/97% vs 78%/86%	Sensitivity: 96% vs 66%	Higher (qualitative)	(69)

Figure caption example:-:No data.

### Peritoneum-dominant tumor

#### Peritoneal cancer

**Figure 2:**
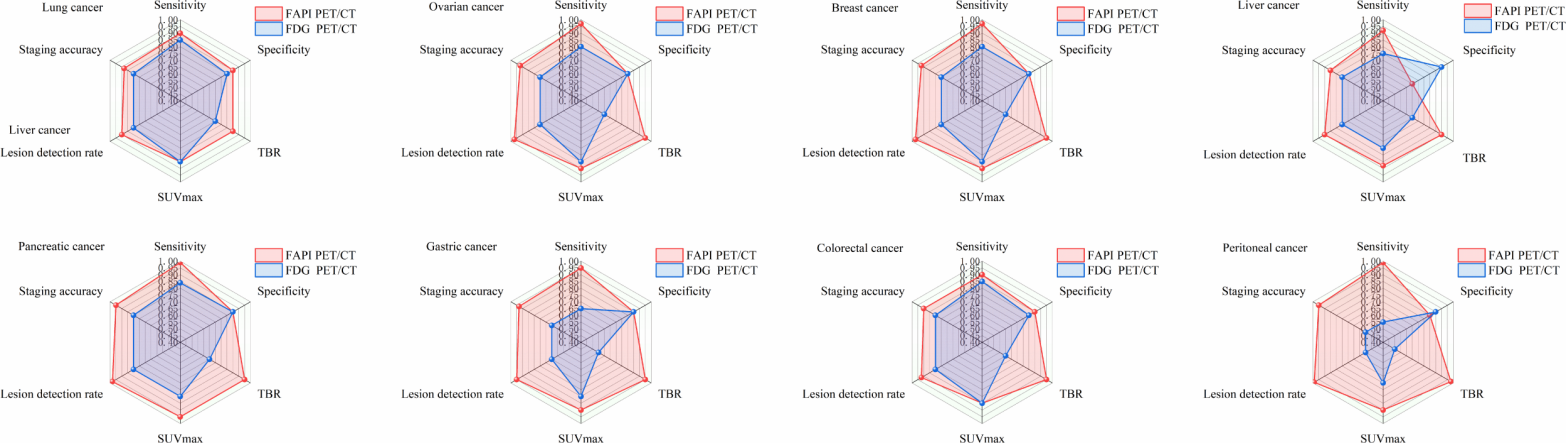
An evidence-weighted radar chart comparison of FAPI PET/CT and ^18^-FDG PET/CT performance across eight major solid tumor types.

Malignant peritoneal surface tumors typically present as diffuse small nodules and serosal dissemination; however, their early detection is challenged by minimal lesion volume, low metabolic activity characteristic of mucinous and signet-ring cell subtypes, and physiological intestinal uptake of tracers (72, 73). By selectively targeting fibroblast activation protein (FAP)-expressing tumor stroma—commonly rich in CAFs and characterized by low physiological bowel uptake—FAPI PET/CT yields superior lesion-to-background contrast compared to ^18^F-FDG PET/CT, especially in mucinous and signet-ring cell carcinomas with low FDG avidity (74, 75). Evidence from a systematic review and meta-analysis (11 studies, 340 patients) confirms this benefit, demonstrating significantly higher pooled patient-level sensitivity for FAPI PET/CT compared with FDG PET/CT (98.2% vs 55.9%), along with a consistent advantage in lesion-level sensitivity (99.9% vs 27.3%) (74). In a paired single-center cohort (n=46), FAPI demonstrated superior sensitivity for detecting peritoneal carcinomatosis (97.67% vs 72.09%); notably, sensitivity in gastric cancer, including the signet-ring cell subtype, improved from 53.85% with FDG to 100% with FAPI, accompanied by a higher median lesion SUVmax (9.82 vs 3.48) and an elevated imaging-derived peritoneal cancer index (18 vs 6) (76). In a prospective paired-cohort study of 113 patients imaged within a two-week interval, FAPI imaging eliminated the need for fasting, utilized a lower administered activity of 0.3–3.1 MBq/kg (compared with 2.8–7.0 MBq/kg for FDG), and demonstrated a favorable safety profile with no adverse events reported. At the patient level, FAPI exhibited superior sensitivity, negative predictive value (NPV), and accuracy relative to FDG (100% vs. 93.2%, 100% vs. 22.22%, and 93.81% vs. 86.73%, respectively), whereas specificity and positive predictive value (PPV) were comparable between the two tracers (77). The minimal physiological intestinal uptake yields a high target-to-background ratio, which enhances the visualization of small nodules located on the mesentery, omentum, and pelvic peritoneal surfaces, thus enabling more accurate assessment of lesion burden and peritoneal carcinomatosis index (PCI) estimation (74, 75). In cases of malignant peritoneal mesothelioma, case-control studies reveal intense fibroblast activation protein inhibitor (FAPI) accumulation (with SUVmax values reaching approximately 20) in areas of peritoneal or omental thickening, even when fluorodeoxyglucose (FDG) uptake remains low to moderate—suggesting a potential diagnostic utility for rare histological subtypes and limited disease burden (78). From a clinical perspective, accurate preoperative prediction of the Peritoneal Carcinomatosis Index (PCI) is critical for patient selection for cytoreductive surgery with hyperthermic intraperitoneal chemotherapy (CRS-HIPEC), as a PCI exceeding 20 often precludes the procedure. Enhancements in imaging sensitivity and tumor-to-background ratio (TBR) could improve the concordance between radiologically assessed PCI and surgical observations, potentially reducing the incidence of non-therapeutic laparotomies. However, prospective multicenter validation and the establishment of standardized image interpretation criteria are imperative (74, 75). Collectively, current evidence indicates that FAPI PET/CT offers superior detection and delineation of peritoneal tumor burden—particularly in FDG-low mucinous and signet-ring cell carcinomas—while forthcoming studies are warranted to further elucidate specificity, pitfalls associated with inflammation-related uptake, and standardization to support broader clinical adoption (74, 76, 77). Radar plots summarize the relative performance of FAPI PET/CT (red) versus ^18^-FDG PET/CT (blue) across six key imaging metrics, including sensitivity, specificity, tumor-to-background ratio (TBR), SUVmax_\text{max}max​, lesion detection rate, and staging accuracy, in eight representative cancer types: lung cancer, ovarian cancer, breast cancer, liver cancer, pancreatic cancer, gastric cancer, colorectal cancer, and peritoneal malignancies. These radar plots are intended as a schematic visualization to highlight tumor-specific diagnostic profiles and complementary strengths of the two tracers, rather than to imply direct numerical comparability or statistical equivalence between metrics (**Figure 2**). Current evidence demonstrates that FAPI-PET/CT is superior to ^18^F-FDG PET/CT in lesion contrast (TBR), diagnostic sensitivity, and accuracy of imaging-based peritoneal cancer index (PCI) assessment for peritoneal malignancies, particularly in low FDG-avid histological subtypes such as mucinous adenocarcinoma and signet ring cell carcinoma, where it provides enhanced detection capability and improved risk stratification. In clinical practice, patients are typically required to fast before ^18^F-FDG PET/CT to minimize physiological glucose uptake in the gastrointestinal tract and ensure optimal image quality. In contrast, FAPI PET/CT generally does not necessitate fasting, offering greater patient convenience and improving adherence to the examination protocol.

#### Other cancers

FAPI-PET/CT demonstrates superior performance in various solid tumors beyond lung, liver, pancreas, ovary, breast, stomach, colorectal, and primary peritoneal malignancies compared to ^18^F-FDG-PET/CT (79).

#### Cholangiocarcinoma

Independent paired studies have consistently demonstrated that FAPI exhibits higher tumor-to-background ratios and greater peak SUV values compared to FDG within the same patient cohort, further substantiating its superior contrast characteristics and enhanced lesion visualization in biliary system tumors (80). Independent paired studies have consistently shown that FAPI achieves higher tumor-to-background ratios and greater peak SUV values compared to FDG in the same patient population, further supporting its enhanced contrast performance and improved lesion visualization in biliary system tumors (81).

#### Nasopharyngeal carcinoma

The lower physiological tracer uptake in the myocardium and liver resulted in enhanced tumor-to-background ratios (TBR) and improved interpretability of mediastinal and perihilar lesions, leading to increased staging accuracy and a reduced risk of false-positive interpretations. In a prospective study of nasopharyngeal carcinoma, FAPI-04 achieved a 100% detection rate for primary tumors, surpassing the 96% detection rate of FDG. Researchers highlighted that the low background activity of FAPI in the head and neck region facilitated clearer delineation of complex anatomical boundaries, thereby improving the accuracy of T staging and local invasion assessment (82).

#### Cervical cancer

Head-to-head data in cervical cancer show no SUVmax difference between FAPI and FDG for primary or metastatic lesions; however, FDG yields more false positives, whereas FAPI reduces misclassification, indicating higher specificity and more reliable lymph-node staging in inflammation-prone pelvic settings. In metabolically cold histologies (e.g., endometrial clear cell carcinoma), FDG uptake is frequently low, while FAPI demonstrates robust avidity in primary and disseminated disease, improving staging; collectively, FAP-targeted stromal imaging increases diagnostic sensitivity and tumor-to-background ratio (80). In addition, early clinical applications have demonstrated that FAPI enables TBR-based quantitative stratification and facilitates imaging-based staging without the need for anesthesia. This technical methodology and its associated quantitative framework lay the groundwork for future standardization efforts and seamless integration with radiotherapy planning protocols (83).

#### Bladder cancer

In a real-world matched cohort analysis of bladder cancer, FAPI demonstrated significantly higher tumor-to-blood pool TBR compared to FDG (P value= 0.001 for inter-lesion TBR comparisons among metastatic sites), and identified a greater number of metastatic lesions within the same patient (31 vs. 22 total lesions). This finding contrasts with the high background signal observed with FDG in the urinary tract, underscoring FAPI’s combined advantages of elevated tracer uptake and reduced background activity. At the individual lesion level, FAPI exhibited significantly higher SUVmax values in hilar lymph nodes and intrapulmonary metastases compared to FDG within the same patients, further emphasizing its enhanced sensitivity for detecting nodules and nodular metastases during systemic staging of urinary tract malignancies (84).

#### Thyroid cancer

In the context of differentiated thyroid cancer, paired comparative analyses have shown that the diagnostic accuracy of ^18^F-FAPI-42 is largely on par with that of FDG. However, FAPI exhibits higher tracer uptake and improved detection rates for local recurrences and lymph node metastases, particularly in FDG-negative lesions and complex anatomical regions, where its low background activity results in superior TBR. In control cases involving radioactive iodine-refractory (RAIR) disease, FAPI showed significantly reduced physiological uptake in high-background organs such as the brain, liver, and myocardium, thereby enhancing the visualization and contrast of small lesions—including pulmonary micrometastases. These findings further support the complementary role of FAPI in “FDG-cold” and RAIR clinical scenarios (85).

#### Head and neck tonsillar carcinoma

Head-to-head evaluation of FAPI-PET/CT demonstrates maintained lesion-background contrast and improved lesion visualization despite the high physiological FDG uptake in the pharyngeal lymphoid ring. This performance aligns with the overall low background distribution of FAPI in the head and neck region and corroborates previous evidence from nasopharyngeal carcinoma (NPC) studies (86).

#### Multiple myeloma

In clinical studies of multiple myeloma, although no statistically significant overall difference in SUVmax was observed between FAPI and FDG for bone lesions, FAPI demonstrated higher SUVmax values in 6 out of 8 individual cases. Moreover, FAPI identified additional bone lesions and medullary cavity involvement in certain patients, features that were not captured by FDG. The potential for FDG to yield false-negative results has been previously reported in the literature. The low background signal associated with FAPI contributes to improved lesion visualization and more comprehensive assessment (87).

#### Lymphoma

FDG remains superior for overall staging and lesion detection. However, FAPI demonstrates added value in specific subregions such as the liver, central nervous system, and head and neck areas, where its low background activity and higher tumor-to-liver ratios (TLR) provide complementary diagnostic information. These findings suggest that FAPI and FDG offer complementary strengths in diverse biological phenotypes and anatomical contexts (88).

#### Dermatofibrosarcoma protuberan

FAPI provides superior visualization of lesion extent and deep tissue invasion compared to FDG, thereby expanding the boundaries of preoperative assessment and potentially influencing surgical planning. This diagnostic advantage aligns with FAPI’s high uptake in tumors characterized by abundant fibrous stroma (89).

#### Epithelioid malignant pleural mesothelioma

FAPI demonstrates superior delineation of diffuse pleural lesions and lymph node involvement at the cardio-diaphragmatic angle compared to FDG. This highlights its advantages in accurately defining lesion boundaries and extent on the pleural surface, particularly in encapsulated (wrapped-type) or metabolically heterogeneous tumors (90).

#### Venous-type leiomyomatosi

A rare yet frequently misdiagnosed solid tumor, exhibits significantly higher uptake of FAPI compared to FDG (as exemplified by SUVmax values of 9.9 vs. 2.0). This indicates FAPI’s strong affinity for lesions composed of smooth muscle and abundant fibrous stroma, offering enhanced capability in tracing systemic metastatic pathways and facilitating more accurate preoperative risk assessment (91–93). However, FAPI is not universally applicable. It also demonstrates sensitivity to fibro-inflammatory processes. Previous studies have reported co-uptake of FAPI and FDG in conditions such as elastofibroma, as well as in spinal and cardiac tuberculosis, which poses challenges to diagnostic specificity. These findings underscore the importance of integrating morphological features, clinical follow-up, and histopathological evidence during image interpretation to prevent over-staging (80, 94).

**Figure 3:**
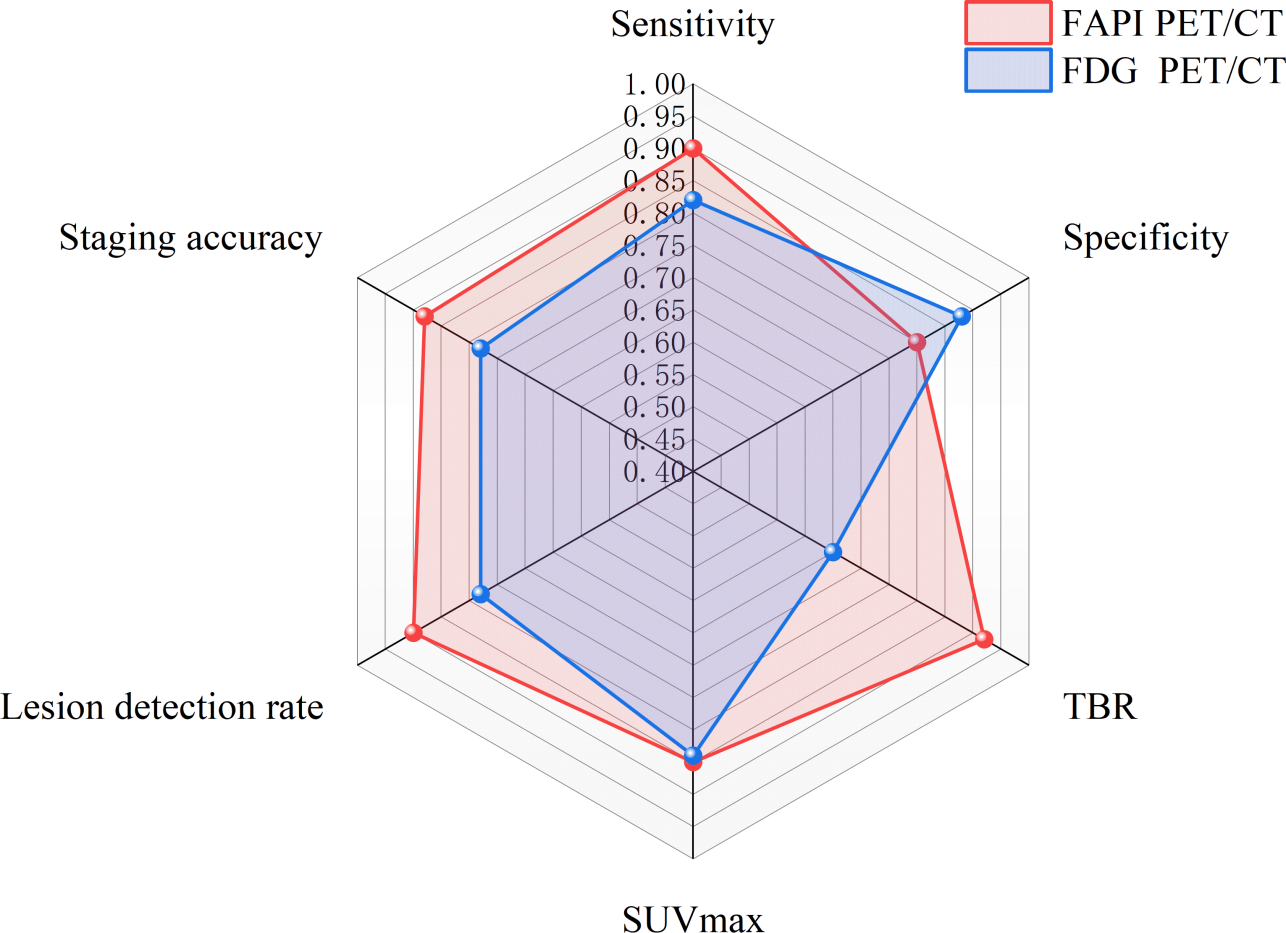
Schematic radar plot summarizing overall performance trends of FAPI PET/CT versus ^18^-FDG PET/CT across solid tumors.

To systematically present the comparative performance of FAPI and ^18^F-FDG across key parameters in other cancers, this article summarizes and synthesizes the available research data (**Table 5**). FAPI PET/CT demonstrates a consistent overall advantage across solid tumors in tumor-to-background ratio, lesion detection rate, and staging accuracy when compared with ^18^-FDG PET/CT. These trends are particularly driven by tumor types characterized by abundant stromal reaction or low glycolytic activity.In contrast, the specificity of FAPI PET/CT appears more variable across tumor types, reflecting its known sensitivity to fibro-inflammatory processes, whereas ^18^-FDG PET/CT maintains relatively stable specificity in selected clinical contexts. Notably, SUVmax shows overall comparable performance between the two tracers, underscoring their complementary biological targets.This schematic summary should be interpreted as an integrative visualization of evidence trends rather than a quantitative comparison (**Figure 3**). The core value of FAPI-PET/CT lies in its role as a complementary rather than replacement imaging modality. It effectively addresses the diagnostic limitations of FDG in tumors with low metabolic activity, regions with high physiological background uptake, and small lesion foci, thereby expanding the armamentarium of clinical imaging tools. However, current evidence is largely derived from single-center or small-sample studies. Future multicenter, prospective trials are essential to establish standardized SUV/TBR threshold criteria and define tumor subtype-specific “application guidelines” Such efforts will facilitate the accurate integration of FAPI-PET/CT into clinical diagnostic and therapeutic pathways, ultimately enabling the translation of its technical advantages into tangible patient benefits.

**Table 5:** Summary of key parameter comparisons (FAPI vs FDG, Other Cancers).

Cancer type	Indicators/Scenarios	Evaluation level	FAPI	FDG	Note	References
Biliary tract tumors	Detection rate of primary lesion	Lesion level	100%	81%	16/16 vs 13/16	(79)
Biliary tract tumors	Detection rate of lymph nodes	Lesion level	98%	83%	41/42 vs 35/42	(79)
Biliary tract tumors	Detection rate of distant metastasis	Lesion level	100%	79%	99/99 vs 78/99	(79)
Esophageal cancer	Lymph node sensitivity	regional level	95.00%	75.00%	–	(95)
Esophageal cancer	Lymph node specificity	regional level	98.40%	77.20%	–	(95)
Esophageal cancer	Accuracy of lymph nodes	regional level	97.30%	76.40%	–	(95)
Esophageal cancer	The mean ± SD of SUVmax of lymph nodes	Lesion level	9.3 ± 5.2	6.4 ± 5.9	–	(95)
Nasopharyngeal carcinoma	Detection rate of primary lesion	Patient level	100%	96%	28/28 vs 27/28	(82)
Nasopharyngeal carcinoma	False positive lymph nodes	Lesion level	2	51	–	(82)
Nasopharyngeal carcinoma	False positive lymph nodes	Lesion level	20	6	–	(82)
Nasopharyngeal carcinoma	The mean ± SD of SUVmax of lymph nodes	Lesion level	11.7 ± 5.0	13.6 ± 5.5	There was no statistically significant difference	(82)
Cervical cancer	Detection rate of primary lesion	Patient level	100%	100%	All 35 were detected.	(81)
Cervical cancer	Detection rate of lymph nodes	Lesion level	100%	98%	50/50 vs 49/50	(81)
Cervical cancer	False positive lymph nodes	Lesion level	0	12	–	(81)
Cervical cancer	The mean ± SD of SUVmax of lymph nodes	Lesion level	7.0 ± 3.5	7.6 ± 4.0	The difference was not significant	(81)
Tonsillar cancer	SUVmax of the primary lesion (mean ± SD)	Lesion level	5.03 ± 4.06	7.90 ± 4.84	Higher FDG	(89)
Tonsillar cancer	Primary lesion TBR (mean ± SD)	Lesion level	3.19 ± 2.06	1.87 ± 1.80	FAPI was significantly higher.(p<0.001)	(89)
Tonsillar cancer	Sensitivity of cervical lymph nodes	Lesion level	87.23%	95.74%	The difference was not significant(p=0.125)	(89)
Tonsillar cancer	Cervical lymph node specificity	Lesion level	46.15%	11.54%	FAPI was significantly higher.(p=0.004)	(89)
Tonsillar cancer	Accuracy of cervical lymph nodes	Lesion level	72.60%	65.75%	FAPI was significantly higher.(p<0.001)	(89)
Bladder cancer	TBR (tumor/blood pool) of metastatic lesion	Lesion level	5.33	1.95	p=0.001	(84)
Differentiated thyroid carcinoma	Sensitivity (patient level)	Patient level	82.80%	96.60%	Higher FDG	(85)
Differentiated thyroid carcinoma	Sensitivity (lesion level)	Lesion level	69.80%	88.60%	Higher FDG	(85)
Differentiated thyroid carcinoma	Positive predictive value (PPV)	Lesion level	73.70%	89.20%	Higher FDG	(85)

Figure caption example:-:No data.

## Current challenges and prospects for future development

FAP is highly expressed on CAFs in most epithelial malignancies, offering a stromal-targeted imaging target that is distinct from glucose metabolism and contributing to the rapid adoption of FAPI PET/CT as a complementary—rather than a complete substitute for—^18^F-FDG PET/CT (18, 96). FAPI imaging capitalizes on the widespread presence and functional contributions of CAFs in tumor invasion, immune modulation, and extracellular matrix remodeling, thereby enhancing lesion visibility even in cases where tumor cell glycolysis is limited (18). Clinical reviews summarize the extensive early experience across various tumor types, highlighting both the potential of FAPI PET/CT and the necessity for standardization (18, 97).

Technically, FAPI tracers frequently achieve higher tumor-to-background ratios compared to FDG, owing to their low physiological uptake in many normal organs, which enhances lesion detectability in the abdomen and central nervous system (CNS) (18, 98). Early human data with ^18^F-FAPI-42 suggest a relatively flexible acquisition window (approximately 60–120 minutes post-injection), characterized by high tumor uptake and generally low standardized uptake values (SUVs) in normal organs, thereby facilitating adaptable imaging workflows (99).

Radiochemistry innovations, such as FAPI-74—which is available as either a ^68^Ga kit or ^18^F-AlF formulation—expand accessibility while maintaining favorable dosimetry profiles (14). In parallel, ^18^F-labeled peptides (e.g., ^18^F-FAP-2286 and ^18^F-AlF-FAP-NUR) demonstrate excellent image quality and offer the advantage of not requiring strict dietary or fasting protocols, thereby facilitating their integration into routine clinical practice (91, 100).

Head-to-head evidence indicates context-dependent performance: in mixed-cancer cohorts, FAPI frequently achieves higher target-to-background ratios with comparable or superior lesion detection compared to FDG, although results may vary by anatomical site and histological subtype (18, 96). In liver metastases ^68^Ga-FAPI detects more lesions than FDG and achieves significantly higher tumor-to-background ratios (TBR), despite occasionally lower absolute lesion SUVmax values, thereby substantially improving lesion conspicuity (101). Meta-analytic synthesis suggests that FAPI outperforms FDG in the detection of nodal metastases, yet demonstrates no significant advantage in the assessment of osseous disease, thereby highlighting its domain-specific diagnostic strengths (102). Disease-specific studies, such as those focused on cholangiocarcinoma, consistently report superior staging performance with FAPI-based imaging compared to FDG and/or CT (80). Prospective studies directly comparing FAPI with FDG for tumor staging are currently underway, reflecting a transition from feasibility assessment to evaluations of clinical decision impact (79, 97).

Biologically, FAPI PET visualizes the tumor microenvironment rather than tumor glycolysis, a mechanistic distinction that enhances sensitivity in desmoplastic cancers while also rendering the modality susceptible to fibroblast-rich benign processes. A systematic review has cataloged numerous non-malignant FAPI-avid conditions—including post-surgical scars, fibrotic or inflammatory lesions, and arthritis—that require clinicoradiologic correlation to prevent over-staging. For example, pancreatitis may exhibit FAPI uptake resembling that of pancreatic cancer, although careful PET/MR evaluation can assist in differentiation. Tuberculosis (including cardiac involvement) can demonstrate avidity for both FDG and FAPI, underscoring the importance of pathologic or microbiologic confirmation in endemic regions (18). Renal findings observed with FAPI imaging—such as cortical signal accumulation in cases of acute kidney injury—highlight how FAP expression during tissue remodeling can generate biologically plausible confounders that pose diagnostic challenges (97, 103).

A promising near-term strategy involves purposeful dual-tracer imaging, which integrates complementary metabolic signals from tumor cells and stromal components to reduce false-negative results and improve tumor phenotyping (79, 102). Single-session FDG+FAPI protocols are feasible and have shown clinically meaningful improvements in staging precision; in esophageal cancer, dual-tracer imaging has influenced radiotherapy target delineation and dose planning decisions (9, 102). Combining PET with MRI takes advantage of MRI’s excellent soft-tissue contrast to improve radiation treatment planning and evaluation of therapeutic response, especially in the pelvic and head-and-neck area (98, 104). Total-body systems can capture whole-organism kinetic data from both tracers, potentially enabling parametric mapping and microdosimetry approaches that correlate imaging phenotypes with clinical outcomes (98).

Theranostics represents another promising frontier: FAPI-based radioligand therapy has entered first-in-human trials (e.g., 90Y/177Lu-FAPI-46), and peptide-based agents such as FAP-2286 provide a genuine imaging-to-therapy continuum (91, 105). Novel chemotypes (e.g., heterobivalent ^68^Ga-FAPI-LM3) aim to achieve stronger binding affinity and enhanced retention, whereas ^18^F-labeling strategies (e.g., ^18^F-FAP-2286, ^18^F-AlF-FAP-NUR) are designed to support mass production and logistical scalability, facilitating broader clinical adoption (100). Forward-looking trials should correlate imaging phenotypes—such as stromal density, nodal versus osseous tropism, and kinetic parameters—with patient-level endpoints, and evaluate how dual-tracer or multimodal imaging strategies influence decisions regarding surgery, radiotherapy, or systemic therapy (9, 96).

In summary, FAPI PET/CT complements ^18^F-FDG by visualizing the stromal component with high contrast and through practical imaging workflows. However, Current barriers to the cost-effectiveness evaluation of FAPI PET/CT include limited large-scale commercial availability of radiotracers, inconsistent production processes and regulatory approvals, substantial heterogeneity among FAPI variants and imaging protocols without established standardization, a scarcity of prospective multicenter cost-effectiveness studies, and an unquantified incremental benefit for clinical decision-making and patient outcomes.the occurrence of biologically plausible false positives and variable performance across different anatomical sites necessitates its careful integration with clinical context, adherence to standardized protocols, and, in many clinical settings, the deliberate use of dual-tracer or multimodal imaging strategies to optimize individualized oncologic care.

## Conclusion

FAPI PET/CT has rapidly evolved into a clinically valuable complementary tool—as opposed to a direct replacement—to ^18^F-FDG, supported by head-to-head comparisons and meta-analytic evidence, and is now formally recognized by the first joint SNMMI/EANM practice guideline for FAP PET (102, 106, 107). In the short term, FAPI’s consistently low physiological background activity in key organs (e.g., brain, liver, oral mucosa) results in high tumor-to-background ratios and lesion conspicuity, thereby facilitating accurate staging and target delineation in scenarios where FDG may be limited by variable normal tissue uptake (106). Optimized protocols (e.g., early post-injection imaging windows) further streamline workflow and may improve lesion detection, while maintaining favorable biodistribution and dosimetric profiles across FAPI-targeted agents (106, 108). Pooled head-to-head analyses demonstrate that FAPI enhances the detection of lymph node metastases across a range of cancer types, with performance for bone metastases being broadly comparable to that of FDG. These findings support the near-term implementation of FAPI PET/CT for nodal mapping and comprehensive cancer staging (102). In parallel, same-session dual-tracer protocols (FDG + FAPI) are technically feasible and demonstrate superior performance compared to single-tracer imaging, enabling comprehensive whole-patient phenotyping by integrating information on glycolysis and stromal activation (109). The biological rationale for FAPI imaging is robust, CAF-rich tumor stroma abundantly expresses FAP, allowing visualization of a microenvironmental hallmark that is distinct from tumor glucose metabolism and thus complementary to FDG (110). Nevertheless, short-term clinical implementation must account for well-documented limitations—FAPI uptake in non-malignant fibroinflammatory conditions (e.g., degenerative bone or joint disease, fibrosis, post-surgical scars, pancreatitis, granulomatous infections) may decrease diagnostic specificity and necessitates careful clinicoradiologic correlation (9, 97, 111). Looking to the long term, the integration of FDG and FAPI into unified, indication-specific imaging algorithms—including single-session dual-tracer protocols—holds promise for enhanced phenotyping to guide response-adaptive therapy, surgical or radiotherapy planning, and the earlier detection of micrometastatic disease (102, 106, 109).

To provide a concise and practice-focused overview, we have consolidated the comparative evidence—organized by disease category, research model, and principal experimental conditions—into a synthesized evidence map (**Table 6**). This table highlights the domains in which FAPI PET/CT exhibits the most consistent added value compared to ^18^F-FDG PET/CT. Current evidence highlights heterogeneity across tumor types and study designs, emphasizing the need for large-scale, prospective, tumor-specific clinical trials that utilize harmonized protocols and quantitative endpoints (102). The near-term value of FAPI PET/CT lies in its ability to improve lesion conspicuity, enhance nodal assessment accuracy, and provide actionable dual-tracer synergy. In the long term, its potential resides in the development of standardized, biology-informed imaging pathways that integrate stromal and metabolic biomarkers to enable personalized cancer care.

**Table 6:** This review provides a summary, organized by disease type, model, and imaging conditions, of the comparative performance between FAPI PET and ^18^F-FDG PET.

Disease	Evidence model	Key imaging conditions	Main conclusions vs FDG
Lung cancer	Prospective real-world + meta-analysis	~1 h p.i.	Improved N/M staging; higher TBR (brain/bone); management impact
Hepatocellular carcinoma	Prospective + retrospective head-to-head	Fibrosis/steatosis may increase background	Superior initial staging, recurrence detection, and small lesion visualization
Ovarian cancer	Prospective H2H + retrospective + systematic evidence	PET/MR for upper abdomen/diaphragm; PCI-based	Advantages in peritoneal disease and small LN; greater impact in recurrence/platinum-sensitive settings
Breast cancer	Prospective H2H + paired analysis	No special preparation	Superior axillary staging; advantages in low-FDG subtypes and brain/bone/liver metastases
PDAC	Prospective H2H + pooled evidence (7 studies/322 pts)	18F-AIF-FAPI-74 with low background	Improved whole-course staging and small lesion detection; future need for multicenter standardization and FDG complementarity
Gastric cancer	Prospective H2H + PET/MR + reviews	≥52% decline in SUVmax/TBR predicts MPR	Superior in FDG-low subtypes and peritoneal metastasis; potential for early response assessment
Colorectal cancer	Multiple H2H + meta-analysis	Low peritoneal/bowel background benefits PCI	Enhanced LN, peritoneal (PCI), and small liver metastasis detection; frequent TNM upstaging
Peritoneal tumors/metastasis	Meta-analysis (11 studies/340 pts) + prospective pairs	No fasting; 0.3–3.1 MBq/kg	High impact on PCI assessment and clinical decision-making; VOI/PCI standardization needed
Cholangiocarcinoma/BTC	Multiple comparative studies	Not specified	Improved lesion conspicuity; prospective stage–outcome validation required
Esophageal cancer	Prospective H2H	Not specified	Reduced overstaging; suitable for RT target delineation and micrometastasis monitoring
Nasopharyngeal carcinoma	Prospective	Low head–neck background	Improved T-stage and invasion assessment; applicable to RT planning and surveillance
Cervical cancer/metabolic-cold gynecologic tumors	Head-to-head studies	Not specified	Increased specificity; improved staging of FDG-low subtypes; threshold standardization warranted
Bladder cancer	Matched-pair real-world analysis	Not specified	Overcomes FDG urinary interference; favorable for systemic staging and micrometastasis detection

## Glossary

^18^F-FDG: Fluorine-18 fluorodeoxyglucose
AUC: Area Under the Curve
CAF(s): Cancer-Associated Fibroblast(s)
CRC: Colorectal Cancer
CRS-HIPEC: Cytoreductive Surgery with Hyperthermic Intraperitoneal Chemotherapy
CT: Computed Tomography
DFSP: Dermatofibrosarcoma Protuberans
DTC: Differentiated Thyroid Cancer
EANM: European Association of Nuclear Medicine
EOC: Epithelial Ovarian Cancer
FAP: Fibroblast Activation Protein
FAPI: Fibroblast Activation Protein Inhibitor
GLUT: Glucose Transporter
GLUT1: Glucose Transporter 1
HCC: Hepatocellular Carcinoma
ICC: Intrahepatic Cholangiocarcinoma
LDCT: Low-Dose Computed Tomography
LN: Lymph Node
LNM: Lymph Node Metastasis
MRI: Magnetic Resonance Imaging
NPC: Nasopharyngeal Carcinoma
NPV: Negative Predictive Value
NSCLC: Non-Small Cell Lung Cancer
OC: Ovarian Cancer
PDAC: Pancreatic Ductal Adenocarcinoma
PET: Positron Emission Tomography
PET/CT: Positron Emission Tomography/Computed Tomography
PET/MR: Positron Emission Tomography/Magnetic Resonance
PPV: Positive Predictive Value
RAIR: Radioactive Iodine-Refractory
ROC: Receiver Operating Characteristic
SNMMI: Society of Nuclear Medicine and Molecular Imaging
SRCC: Signet Ring Cell Carcinoma
SUV: Standardized Uptake Value
SUVmax: Maximum Standardized Uptake Value
TBR: Tumor-to-Background Ratio
TLR: Tumor-to-Liver Ratió
TME: Tumor Microenvironment
TNM: Tumor–Node–Metastasis
VOI: Volume of Interest.
